# Obstetric and perinatal outcomes of intracytoplasmic sperm injection for infertile men with Y chromosome microdeletions

**DOI:** 10.1097/MD.0000000000017407

**Published:** 2019-10-11

**Authors:** Qi Xi, Zhihong Zhang, Ruixue Wang, Linlin Li, Leilei Li, Haibo Zhu, Ruizhi Liu, Lili Luo

**Affiliations:** Centre for Reproductive Medicine, Centre for Prenatal Diagnosis, First Hospital, Jilin University, Changchun, China.

**Keywords:** AZF microdeletion, ICSI, neonatal outcome

## Abstract

**Background:**

To evaluate the safety of intracytoplasmic sperm injection (ICSI) for men with Y chromosome azoospermia factor (AZF) microdeletions.

**Methods:**

Twenty-five men with Y chromosome microdeletions and their partners underwent ICSI treatment. These subjects were matched against 50 ICSI cycles in which the patients had normal Y chromosomes.

**Results:**

Among the 25 couples, 17 achieved a clinical pregnancy of which 14 continued to a live birth. Sixteen men had deletions of AZFc markers (sY152, sY254, and sY255), 1 had a deletion of sY152, 3 had a deletion of sY254, sY255, 1 had a deletion of sY152, sY239, Sy242, sY254, and sY255, and 3 had deletions of sY152, sY254, sY255, and sY157. AZFb microdeletions (sY127, sY134, and sY143) were found in 1 patient. AZF microdeletions had no adverse effects on the clinical pregnancy, implantation or delivery rates, birth weight, gestational age, or sex ratio when compared with the control group. Overall, the multiple gestation and preterm delivery rates of the AZF microdeletion group were similar to those in the control group.

**Conclusion:**

Men with AZF microdeletions can achieve the delivery of healthy children using ICSI. In this series, it produced good implantation rate and obstetric and perinatal outcomes.

## 1 Introduction

About 10% to 15% couples of reproductive age experience clinical infertility.^[1]^ Half of these cases involve male factors,^[2]^ and up to 30% of cases of male infertility arise from genetic defects that can cause sperm production disorders.^[3]^ The azoospermia factor (AZF) region of the Y chromosome plays a vital role in the genetics of male infertility. Most Y chromosome microdeletions occur on the long arm of the Y chromosome (Yq11) and are subdivided into 3 AZF regions: a, b, and c. These genes are involved in spermatogenesis, and microdeletions in this region lead to spermatogenetic defects.^[4]^

The development of assisted reproductive technologies (ARTs) such as intracytoplasmic sperm injection (ICSI) and microdissection testicular sperm extraction (micro-TESE) have resulted in increasing numbers of men with Y chromosome microdeletions having the opportunity to become fathers. However, such paternal microdeletions of the Y chromosomes can be transmitted through ICSI, which is a concern for the future fertility of their sons.^[5]^ It is necessary to investigate whether Y chromosome microdeletions can bring about any adverse effects on the resulting babies, such as increased rates of congenital birth defects. Here, we aimed to evaluate the effects of AZF microdeletions on the obstetric and perinatal outcomes of cycles using ICSI for male infertility treatment.

## 2 Materials and methods

This is a comparative study that received institutional review board approval from Medical Ethics Committee of First Hospital of Jilin University (2013-264) and written consent was obtained from the patients.

### 2.1 Patients

From March 2013 to November 2016, we reviewed 25 men with Y chromosome microdeletions who underwent ICSI cycles with their partners at the Center for Reproductive Medicine, First Hospital of Jilin University, P.R. China. The control group involved 50 in vitro fertilization (IVF)/micro-TESE cycles during the same period in which the men had normal Y chromosomes but presented with oligozoospermia or nonobstructive azoospermia. Control couples were matched for infertility duration, female body mass index, female age, male age, numbers of oocytes retrieved, numbers of metaphase II oocytes produced, and numbers of fetuses produced.

### 2.2 Y chromosome analysis

Genomic DNA was isolated from peripheral blood cells collected in ethylene diamine tetraacetic acid-coated tubes. A multiplex polymerase chain reaction technique was applied for Y chromosome analysis. Single primer pairs confirmed the absence of each site in a polymerase chain reaction on multiple occasions as failure to amplify a sequence-tagged site (STS) on the Y chromosome. The STS markers were as follows: sY84, sY86, sY127, sY134, sY143, sY152, sY157, sY239, sY242, sY254, and sY255. The STS markers sY14, Y-linked zinc finger protein, and X-linked zinc finger protein were used as internal controls.

### 2.3 Clinical procedures and embryo culture

All patients underwent micro-TESE/IVF/ICSI according to standard protocols. Cycles were generally initiated using a monthly oral contraceptive pill. Then all women underwent a luteal phase gonadotropin releasing hormone stimulation protocol with step-up gonadotropin dosing. Human chorionic gonadotropin (5000 or 10,000 IU) was administered to induce ovulation when 3 or more follicles were >15 mm in diameter by ultrasonographic monitoring with the lead follicle being at least 18 mm. Oocytes retrieval was performed 36 to 37 hours after the human chorionic gonadotropin injection. Oocytes selected for IVF or ICSI were preincubated for 2 to 3 hours.

Fertilization and embryo culture were performed in Quinn's-1020 medium enriched with 5% human serum albumin and Quinn's-1026 medium with 10% serum protein substitute, respectively, at 37 °C under 5% CO_2_ in humidified air. The blastocyst culture medium used was Quinn's-1029 medium supplemented with 10% serum protein substitute incubated at 37 °C under 6% CO_2_, 5% oxygen, and 89% N_2_. All media and supplements were from SAGE BioPharma, Bedminster, NJ. Embryos or blastocysts were transferred to recipients using a catheter (Sydney IVF Embryo Transfer Set; Cook Medical, Brisbane, QLD, Australia) with ultrasound guidance.

### 2.4 Outcome measures

Maternal age (in years) was calculated at the time of preparing the micro-TESE/IVF/ICSI cycles. Clinical pregnancies were subsequently evaluated for the presence of fetal heartbeat at 6 to 8 weeks of pregnancy by ultrasound. The live birth rate was calculated as birthing events per embryo transfer (where the birth outcome was known). The miscarriage rate was calculated from the number of fetal heartbeat-positive pregnancies that did not result in a live birth (where the birth outcome was known). The neonatal outcome data were obtained by telephone interview of the parents after delivery. Information was obtained on neonatal gender, birth weight, weeks of gestation, and any congenital birth defects.

### 2.5 Statistical analysis

Data are presented as the mean ± standard deviation, medians, and percentages. All calculations and analysis were carried out using SPSS 17.0 software (SPSS Inc., Chicago, IL). Pearson Chi-squared test, Student *t* test, or the Mann–Whitney nonparametric *U* test were used to determine any statistical differences between means or groups, and *P* < .05 was considered statistically significant.

## 3 Results

The most frequent microdeletions of the patients were detected in the AZFc region, as shown in Table 1. The couple's clinical characteristics in the AZF microdeletion and control groups are listed in Table 2. There were no significant differences between these groups in the duration of infertility, female body mass index, female age, or male age, respectively.

**Table 1:**
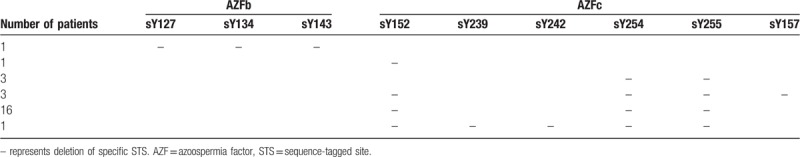
Pattern of STS deletions in the 25 infertile men with chromosome Y microdeletions.

**Table 2:**
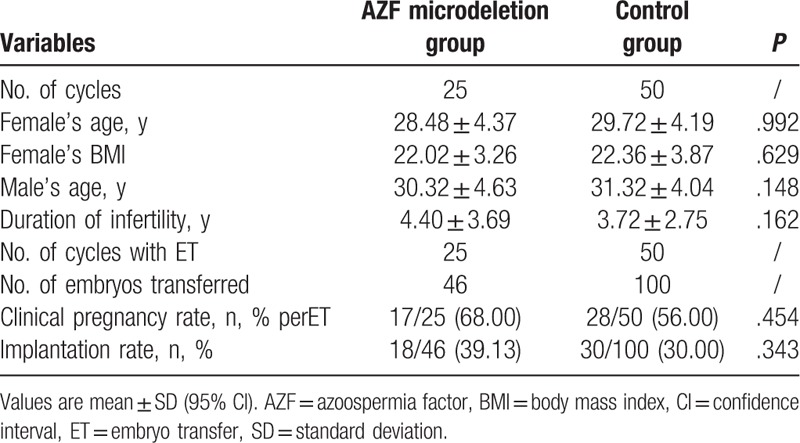
Clinical characteristics of AZF microdeletion and control group.

Table 3 shows the clinical outcomes of the AZF microdeletion and control groups. Seventeen couples in the AZF microdeletion group achieved clinical pregnancies. The respective implantation rates were similar in the 2 groups (39.13% vs 30.00%, *P* = .343). The clinical pregnancy rate was higher in the AZF microdeletion group than in the control group (68.00% vs 56.00%, *P* = .454), but this was not significantly different. No miscarriages or ectopic pregnancies occurred in the AZF microdeletion group. Abnormal gestations and births were 2 embryo damages, 1 induced abortion, and 1 newborn baby in AZF microdeletion group died due to pulmonary undeveloped. Fourteen set live deliveries included in 13 singleton and 1 twins from a set.

**Table 3:**
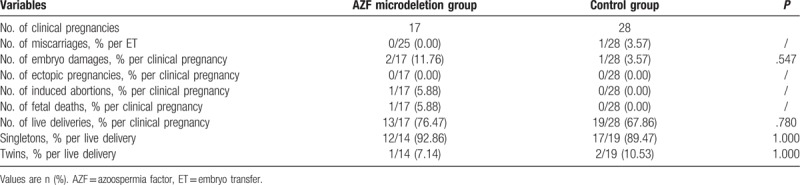
Clinical outcomes of AZF microdeletion and control group.

The partners of the 14 infertile men with chromosome Y microdeletions got live birth. Fourteen cases got delivery of live birth, 8 had deletions of AZFc markers (sY152, sY254, and sY255), 2 had a deletion of sY254, sY255, and 3 had deletions of sY152, sY254, sY255, and sY157. AZFb microdeletions (sY127, sY134, and sY143) were found in 1 patient (Table 4).

**Table 4:**
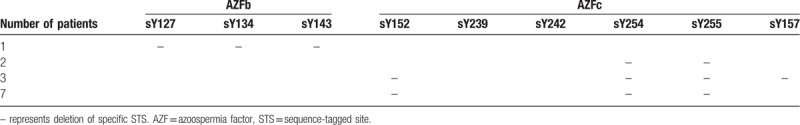
Pattern of STS deletions in the 13 infertile men with chromosome Y microdeletions getting live birth.

The neonatal and perinatal outcomes of the AZF microdeletion and control group are shown in Table 5. No congenital birth defects occurred in the AZF microdeletion group. There were significantly differences in mean gestational age, delivery method, and preterm deliveries between the AZF microdeletion and control group. There were 15 babies (8 male and 7 female) born from the AZF microdeletion embryos, with a mean birth weight of 3171.80 ± 601.65 g, which was similar with deliveries from the control group (3156.19 ± 689.82 g) (*P* = .406). The AZF microdeletion group had a higher ratio of male to female babies (1.14) than the control group (0.91), but the difference was not significant.

**Table 5:**
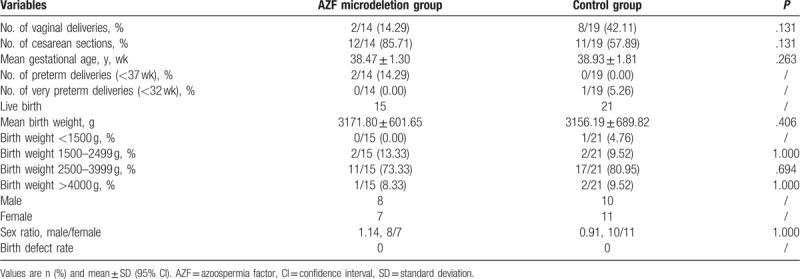
Neonatal outcomes of AZF microdeletion and control group.

## 4 Discussion

The AZF microdeletion in the Y chromosome is considered the second most common cause of spermatogenic arrest, after Klinefelter syndrome.^[6]^ This genomic region is involved in spermatogenesis and is the most common microdeleted region in infertile men.^[7]^ AZFc or AZFb deletions were found in these patients with azoospermia or severe oligozoospermia. One case with AZFb microdeletions and all of AZFc deleted was included in this study. The other 24 infertile men had partial AZFc microdeletions. We reported previously that 38.5% of Y chromosome abnormality carriers had AZF microdeletions, and most were observed in those with a 46,X,Yqh karyotype.^[8]^ Previous studies have revealed similar clinical features in patients with or without Y chromosome microdeletions.^[9,10]^ However, the obstetric and perinatal outcomes for infertile couples where the man has AZF microdeletions have seldom been reported.

In our findings, once spermatozoa were obtained from men with Y microdeletions, the live delivery rate was not significantly different from the results for men without Y microdeletions. Few spermatozoa are required for ICSI treatment and embryologists usually select those with normal morphology and good motility. This could have explained why the clinical pregnancy outcomes were not affected by the sperm source. The clinical pregnancy rate in the Y microdeletion group was 12% higher than in the control group, but our small sample size (n = 25) might have affected this.

In our study, although the differences were not statistically significant, there was a higher male/female ratio of neonates in the Y microdeletion group (8:7) than the control group (10:11). That is to say, AZF microdeletions were not consistent with affecting Y-bearing spermatozoa. We need to increase case numbers to confirm this. As this came from a single-center study with and a small case number, we would like to unite multiple centers and enlarge the sample size for further study.

Men with Y chromosome microdeletions and azoospermia or severe oligozoospermia now have the opportunity to reproduce by using ART. Some studies^[5,11]^ have claimed that male offspring carrying Y microdeletions can result from ART attempts, especially when using micro-TESE to increase the chances of recovering spermatozoa.^[12]^ All male offspring have risks of inheriting Y chromosome defects from their fathers. Although inheritance of the AZFc microdeletion seemingly has no somatic effect on sons,^[12]^ the fear remains that the transmission of microdeletions from father to son will confer adverse effects on male fertility. Preimplantation genetic diagnosis should be provided to all such couples.

Some studies have claimed that AZF microdeletions might be connected causally with miscarriage.^[13,14]^ However, in our study, there were no significant differences in miscarriage rates between the 2 groups and no miscarriage, ectopic pregnancy, or congenital birth defects occurred in the AZF microdeletion group. These findings further suggest that AZF microdeletions do not affect the obstetric and perinatal outcomes after ICSI.

## 5 Conclusions

Patients with AZF microdeletions can achieve good clinical pregnancy outcomes using ICSI. The neonatal and perinatal outcomes for ICSI were similar between patients with or without Y chromosome AZFc microdeletions. This suggests that such microdeletions confer no adverse effects on ICSI outcomes. Our findings indicate that ICSI should be offered to patients with an AZFc deletion and that oligozoospermic patients with AZFb microdeletions are likely to become a father successfully.

## Acknowledgments

The authors thank all the staff of the Genetics Laboratory and IVF Laboratory for their excellent work. All authors have read and approved the final manuscript. The authors also thank James Cummins, PhD, from EDANZ Group (www.edanzediting.com/ac) for editing a draft of this manuscript.

## Author contributions

**Data curation:** Ruixue Wang, Linlin Li.

**Funding acquisition:** Zhihong Zhang.

**Methodology:** Leilei Li, Haibo Zhu.

**Project administration:** Ruizhi Liu.

**Resources:** Ruizhi Liu.

**Software:** Haibo Zhu.

**Writing – original draft:** Qi Xi, Zhihong Zhang.

**Writing – review & editing:** Lili Luo.
